# From Junk to Function: LncRNAs in CNS Health and Disease

**DOI:** 10.3389/fnmol.2021.714768

**Published:** 2021-07-19

**Authors:** Rafaela Policarpo, Annerieke Sierksma, Bart De Strooper, Constantin d’Ydewalle

**Affiliations:** ^1^VIB-KU Leuven Center For Brain & Disease Research, Leuven, Belgium; ^2^Laboratory for the Research of Neurodegenerative Diseases, Department of Neurosciences, Leuven Brain Institute (LBI), KU Leuven, Leuven, Belgium; ^3^Neuroscience Discovery, Janssen Research & Development, Janssen Pharmaceutica N.V., Beerse, Belgium; ^4^UK Dementia Research Institute, University College London, London, United Kingdom

**Keywords:** long non-coding RNAs, central nervous system, neuronal development, neurological disorders, gene regulation

## Abstract

Recent advances in RNA sequencing technologies helped to uncover the existence of tens of thousands of long non-coding RNAs (lncRNAs) that arise from the dark matter of the genome. These lncRNAs were originally thought to be transcriptional noise but an increasing number of studies demonstrate that these transcripts can modulate protein-coding gene expression by a wide variety of transcriptional and post-transcriptional mechanisms. The spatiotemporal regulation of lncRNA expression is particularly evident in the central nervous system, suggesting that they may directly contribute to specific brain processes, including neurogenesis and cellular homeostasis. Not surprisingly, lncRNAs are therefore gaining attention as putative novel therapeutic targets for disorders of the brain. In this review, we summarize the recent insights into the functions of lncRNAs in the brain, their role in neuronal maintenance, and their potential contribution to disease. We conclude this review by postulating how these RNA molecules can be targeted for the treatment of yet incurable neurological disorders.

## Introduction

The sequencing of the ∼3.1 billion base pairs of the human genome marked the completion of the Human Genome Project in April 2003 after a little over 10 years of research. One of the main findings of this large scale project was that only a fraction of the genome encoded in total ∼22,300 protein-coding genes, whereas the remaining fraction was considered “junk DNA” (Human Genome Project, 2003). Since then, our perception of the complexity of the human genome changed dramatically (Frankish et al., 2021). In the past 20 years, advances in RNA sequencing technology pointed out that approximately 80% of our genome is actually transcribed, whereas only around 2% is subsequently translated into proteins (Carninci et al., 2005; Dunham et al., 2012; Hon et al., 2017; Zhao et al., 2021). Besides protein-coding messenger RNAs (mRNAs), transfer RNA (tRNAs) and ribosomal RNAs (rRNAs), other essential RNA species include long non-coding RNAs (lncRNAs), circular RNAs (circRNAs), and small non-coding RNAs (sncRNA) such as microRNAs (miRNAs) and small-interfering RNAs (siRNAs) (Mattick and Rinn, 2015; Quan et al., 2017; Lorenzi et al., 2021).

**FIGURE 1:**
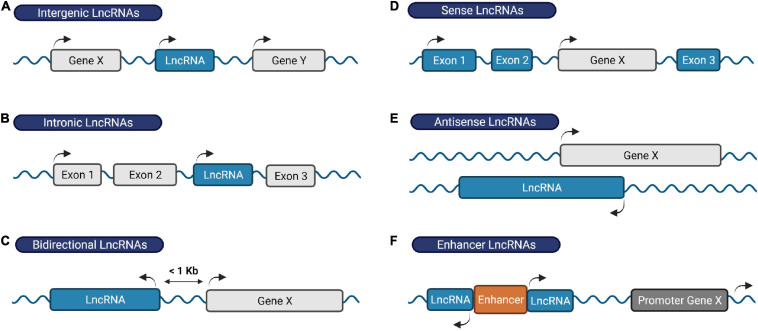
Classification of lncRNAs based on their genomic location. **(A)** Intergenic RNAs are located between two protein-coding genes, **(B)** intronic lncRNAs are transcribed within an intronic region of a protein-coding gene, **(C)** bidirectional lncRNAs are located on the opposite strand of a protein-coding gene whose transcription initiates less than 1,000 base pairs away, **(D)** sense lncRNAs are transcribed from and overlap with the sense strand of a protein-coding gene, **(E)** antisense lncRNAs originate from the antisense strand of a protein coding-gene, and **(F)** enhancer RNAs derive from enhancer regions and play a role in gene transcription activation. Protein-coding genes/exons shown in gray; LncRNA genes/exons shown in blue.

Remarkably, the majority of our DNA generates a large number of lncRNAs (Iyer et al., 2015; Bonetti and Carninci, 2017; Yao et al., 2019; Zhao et al., 2021). By definition, lncRNAs contain more than 200 nucleotides in length and lack protein-coding potential. Based on their genomic location and orientation relative to neighboring protein-coding genes, lncRNAs are broadly categorized into intergenic lncRNAs, intronic lncRNAs, bidirectional lncRNAs, sense lncRNAs, antisense lncRNAs and enhancer RNAs (Figure 1) (Ma et al., 2013; Youse et al., 2020).

LncRNA promoters are structurally similar to those from protein-coding genes, exhibiting characteristic profiles of transcriptional activity markers (e.g., histone acetylation, methylation, ubiquitination, etc.) around their transcription start sites (Derrien et al., 2012; Djebali et al., 2012; Roberts et al., 2014). Consistent with their promoter structure, lncRNAs are generally transcribed by RNA polymerase II (Hon et al., 2017). Additionally, many lncRNAs share some features of messenger RNAs (mRNAs) at the transcript level including the presence of a 5′-cap, 3′-polyadenylation, and the occurrence of alternative splicing events that give rise to alternative transcript isoforms (Derrien et al., 2012; Djebali et al., 2012; Roberts et al., 2014). The mechanisms that regulate these post-transcriptional events in lncRNAs are not well documented; the limited amount of evidence available suggests that the same mechanisms are used as for protein-coding genes albeit with different efficiency (Gil and Ulitsky, 2018; Statello et al., 2021). There are also marked differences between lncRNAs and protein-coding genes. LncRNAs are generally expressed at lower levels although there are examples of highly expressed lncRNAs (e.g., *TUG1* and *MALAT1*) (Derrien et al., 2012; Washietl et al., 2014; Jiang et al., 2016). LncRNAs also exhibit a more highly specific spatiotemporal expression pattern, and display poorer sequence conservation across species compared to protein-coding genes (Derrien et al., 2012; Djebali et al., 2012; Jiang et al., 2016). The high level of orchestrated regulation at the transcriptional and post-transcriptional level suggests that lncRNAs are at least as functionally relevant as protein-coding genes despite low conservation and expression levels.

The lncRNA *Xist* (X-inactive-specific transcript) was one of the first lncRNAs identified when it was isolated in the early 1990s from a female placental complementary DNA library screening (Brown et al., 1991). This lncRNA remains the most studied nuclear lncRNA to date. *Xist* is exclusively expressed from one of the two X chromosomes in females during early embryonic development. The transcript “*coats*” the X chromosome in *cis* and triggers a series of events that results in chromosome-wide transcriptional silencing and heterochromatinization acting in concert with many other lncRNAs, RNA binding proteins and DNA (Rocha and Heard, 2017). As such, *Xist* ensures X chromosome inactivation and dosage compensation between females and males in mammals (Brown et al., 1991).

*Xist* is an archetypical example on the functional relevance of lncRNAs. Evolutionary biologists uncovered that the amount of transcribed DNA (as lncRNAs) correlates with the organisms’ complexity and genome size (Rubin et al., 2000; Mattick, 2001; Taft et al., 2007; Liu et al., 2013; Kapusta and Feschotte, 2014). These observations further suggest that despite low sequence conservation across species, lncRNAs may contribute to the development of complex organisms and organs including the central nervous system (CNS) (Aprea and Calegari, 2015). In this review, we will summarize the current knowledge on the role of lncRNAs in brain physiology and disease; and discuss the future perspectives and challenges of using lncRNAs as RNA-based therapeutic targets in neurological disorders.

## LncRNAs in the CNS

**FIGURE 2:**
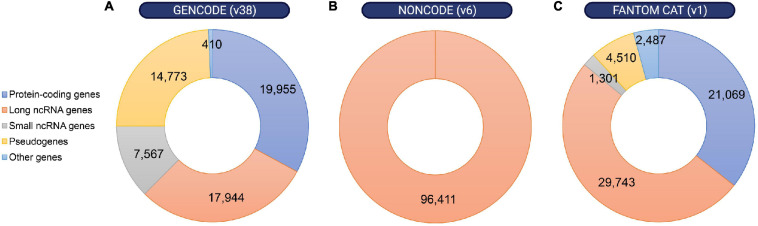
Distribution of annotated loci in the human genome according to different gene map databases. Despite the discrepancies between different annotation methods, non-coding RNA loci are remarkably abundant within the human genome. Number of protein-coding genes, long or small non-coding RNA genes, pseudogenes and other genes were obtained from **(A)** GENCODE release 38 (https://www.gencodegenes.org/human/stats.html), **(B)** NON-CODE v6.0 (http://www.noncode.org/analysis.php), and **(C)** FANTOM CAT v1 (https://fantom.gsc.riken.jp/cat/v1/#/genes).

The observation that most of the mammalian genome is actively transcribed, that much of this pervasive transcription is likely functional, and that lncRNA loci are linked to key biological functions has boosted the interest in lncRNAs (Dunham et al., 2012; Kopp and Mendell, 2018; Lorenzi et al., 2021; Statello et al., 2021; Zhao et al., 2021). However, the first challenge in understanding the physiological relevance of lncRNAs is the difficulty to annotate these transcripts (Uszczynska-Ratajczak et al., 2018). The development of recent sequencing technologies has been key for the emergence of different annotation methods and databases that continue to improve our knowledge of these genes. A few examples are the GENCODE geneset, which combines manual with automated annotation from two pipelines (Ensembl-HAVANA and Ensembl-Genebuild) for protein-coding genes annotation, but uses mostly manual annotation for lncRNA genes (Frankish et al., 2021); NONCODE, an integrative database exclusively dedicated to the annotation of non-coding RNAs, in particular lncRNAs in animals (Zhao et al., 2021); or FANTOM CAT, a meta-assembly mostly based on CAGE (Cap Analysis of Gene Expression) data and annotations from diverse sources (Figure 2) (Hon et al., 2017). Thus, while the number of functional lncRNAs is still a matter of debate, between 15,000 and 100,000 lncRNAs have recently been reported to exist in the human genome. Similar numbers have been annotated in the mouse genome (Uszczynska-Ratajczak et al., 2018). In comparison, approximately 20,000 protein-coding genes have been mapped (Salzberg, 2018). Thus, given their relative quantities, it is hypothesized that lncRNAs contribute to the organism’s complexity.

The development and maintenance of the nervous system in particular is a complex process that must rely on highly coordinated spatiotemporal gene expression programs to finetune the balance between cell proliferation and differentiation and ensure these cell populations give rise to a functional network. A remarkable amount of 40% of all annotated tissue-specific lncRNA genes are specifically enriched in particular brain regions or cell types and participate in many aspects of brain function and development (Derrien et al., 2012; Francescatto et al., 2014; Briggs et al., 2015; Zimmer-Bensch, 2019). In this section, we will discuss some features of lncRNAs that support a widespread functional role for this RNA subclass in mediating CNS development and function.

### Brain LncRNAs Are Evolutionary Conserved

Accumulating data suggest that conserved lncRNAs are more likely to be functionally relevant. While lncRNAs exhibit higher sequence conservation in their promoter regions, splice-junction motifs and small functional domains, lncRNA gene bodies exhibit an overall lower evolutionary conservation relative to protein-coding genes (Chodroff et al., 2010; Ulitsky et al., 2011; Derrien et al., 2012; Necsulea and Kaessmann, 2014; Nitsche et al., 2015). A possible explanation is the fact that many lncRNAs have a recent evolutionary origin, and approximately one third of human lncRNAs has been reported to be restricted to the primate lineage (Derrien et al., 2012; Necsulea and Kaessmann, 2014; Washietl et al., 2014). Accordingly, these lncRNAs seem to have less exonic sequence constraints compared to ancestral and conserved lncRNAs (Necsulea and Kaessmann, 2014; Washietl et al., 2014). Moreover, several lncRNAs have been shown to be under positive evolutionary selection in human compared to other mammalian species (Tay et al., 2009; Lindblad-Toh et al., 2011; Grossman et al., 2013; Francescatto et al., 2014; Washietl et al., 2014; Sarropoulos et al., 2019).

Nonetheless, brain-specific lncRNAs display the strongest evolutionary conservation compared to those expressed in other tissues (Ponjavic et al., 2009; He et al., 2014), and their levels of sequence conservation correlate very well with the levels of brain complexity (Johnson et al., 2015). Intergenic lncRNAs that show strong evolutionary conservation (when evaluating mouse and human genomes) are particularly enriched in the brain (Ponjavic et al., 2009). Moreover, these enriched lncRNAs are located adjacent to protein-coding genes that are co-expressed in the brain and involved in transcriptional regulation or CNS development (Ponjavic et al., 2009).

Other studies indicate that some lncRNAs with relevant functional roles in the brain have been subjected to higher sequence constraints throughout evolution. For instance, the lncRNAs *linc-Brn1b*, *Dali*, and *Pnky* are 3 conserved lncRNAs located in close proximity of and co-expressed with *Brn1* and *Brn2* (Sauvageau et al., 2013; Chalei et al., 2014; Ramos et al., 2015). These two genes encode the POU-homeodomain transcription factors POU3F3 and POU3F2, respectively, that play crucial roles in neocortex development (Sugitani et al., 2002). *Linc-Brn1b* and *Dali* are located downstream of *Brn1* and loss-of-function studies demonstrated that these lncRNAs are required for correct brain development (Sauvageau et al., 2013; Chalei et al., 2014). *Pnky* is a lncRNA transcribed in the opposite strand of *Brn2* with two regions conserved in vertebrates, and its expression is upregulated in neural stem cells in the developing mouse and human cortex. *Pnky* controls neuronal differentiation in dividing neural stem cells by interacting with PTBP1, a splicing factor that represses the inclusion of neural exons in non-neural cells (Ramos et al., 2015). Additional examples of well-conserved lncRNA genes with a role in neurogenesis include the lncRNA *Rmst* and *Tuna*; knockdown of these lncRNAs suppresses neuronal differentiation in mouse embryonic stem cells (Ng et al., 2013; Lin et al., 2014).

Structural features rather than the primary sequence can contribute to functional relevance of lncRNAs (Torarinsson et al., 2006; Seemann et al., 2012; Smith et al., 2013; Diederichs, 2014; Johnsson et al., 2014; Kapusta and Feschotte, 2014). For example, the lncRNA *MALAT1* contains a large number of helices that are highly conserved across vertebrates and even more in mammalian *MALAT1* homologs, indicating the presence of an evolutionary conserved core with predicted functional roles in mammals and vertebrates (McCown et al., 2019).

### LncRNA Genes Exhibit Highly Specific Expression Patterns in the CNS

The CNS is one of the most complex organs in mammals, exhibiting a tight spatial and temporal organization and gene expression profile during development and homeostasis. Several studies showed that lncRNA expression is spatially more restricted than mRNAs in multiple brain regions suggesting that lncRNAs may play crucial roles in the coordination of the complex gene expression in distinct CNS regions (Belgard et al., 2011; Luo et al., 2013; Ramos et al., 2013; Kadakkuzha et al., 2015).

A systematic *in situ* hybridization analysis demonstrated that the expression of the majority of lncRNAs in the mouse brain is restricted to distinct neuroanatomical loci (Mercer et al., 2008). The lncRNA *MIAT*/*Gomafu* was identified as a nuclear retained lncRNA that is specifically expressed in a distinct set of postmitotic neurons within the mouse nervous system (Sone et al., 2007). *Gomafu*-deficient mice develop normally but demonstrate mild hyperactivity associated with an increase in dopamine levels specifically in the nucleus accumbens (Ip et al., 2016). Recently, *Abhd11os* (*ABHD11-AS1* in human) has been identified as a striatal-specific lncRNA and proposed to play a neuroprotective role against mutant Huntingtin (HTT), a protein implicated in Huntington’s Disease (Francelle et al., 2015). Studies combining bulk tissue RNA sequencing with single-cell RNA sequencing approaches confirmed cell-type and region specificity (Liu et al., 2016; Fan et al., 2020).

Taken together, expression analyses and functional studies have demonstrated that the spatial expression of a large number of lncRNAs is tightly regulated in various brain regions. However, the mechanisms that control this restricted expression pattern are not yet fully understood.

### LncRNAs Are Dynamically Regulated in the CNS

The expression of many lncRNAs is temporally regulated during CNS development and several of them are linked to the regulation of protein-coding genes that play crucial roles in neurodevelopment (Briggs et al., 2015). Expression profiling of both protein-coding and non-coding transcripts during differentiation of mouse embryonic forebrain-derived neural stem cells indicated that 5% of approximately 3,600 analyzed lncRNAs are differentially expressed during neuronal-glial fate specification and oligodendrocyte lineage maturation (Mercer et al., 2010). Many of these lncRNAs exhibit coordinated expression with protein-coding genes involved in neuronal and glial lineage differentiation at distinct developmental stages, suggesting that these ncRNAs might regulate the expression of their associated protein-coding genes (Mercer et al., 2010).

Several reports also demonstrated that lncRNA expression is thoroughly regulated in processes such as synaptogenesis and in response to neuronal activity and plasticity (Zalfa et al., 2003; Bernard et al., 2010; Barry et al., 2014). For instance, the lncRNA *ADEPTR* is upregulated in an activity-dependent manner and consequently transported to synapses where it modulates the structural plasticity of dendritic spines in mouse hippocampal neurons (Grinman et al., 2021). Keihani et al. (2019) also identified *NeuroLNC* as a nuclear and neuron-specific lncRNA involved in the regulation of genes with crucial roles in neuronal physiology including neurotransmitter release, synapse organization and neuronal migration in rat models.

Is the expression of lncRNAs also temporally regulated in the human CNS? Lipovich et al. (2014) profiled the expression of thousands of lncRNAs in the human neocortex using microarray technology and identified 8 lncRNA genes with specific developmental expression patterns. The majority of these lncRNAs are located antisense and/or close to known protein-coding genes. Moreover, these loci exhibit primate-specific gene structure features. A transcriptomic analysis of human neurons derived from induced pluripotent stem cells revealed the presence of more than 1,500 lncRNAs whose expression is regulated during their transition to early differentiating neurons (Lin et al., 2011). Many of these lncRNAs are associated with chromatin-remodeling complexes such as RE1-Silencing Transcription factor (REST), REST corepressor 1 (CoREST) and Polycomb Repressive Complex 2 (PRC2) (Lin et al., 2011).

Widespread changes in the transcriptome, including in the expression of lncRNAs, also occur during aging (Wood et al., 2013; Barry et al., 2015; Kour and Rath, 2015; Chen et al., 2017). Several studies demonstrated that lncRNAs can be either up- or downregulated in senescent cells. These lncRNAs are typically associated to protein-coding genes that play a role in cell cycle arrest, cellular growth/tumor suppression, telomere organization and p53 signaling (Grammatikakis et al., 2014; Quinodoz and Guttman, 2014; Angrand et al., 2015; Xu C. L. et al., 2020). These molecular functions all have a clear link to aging. Finally, genome-wide association studies identified single-nucleotide polymorphisms associated with neurological diseases including Schizophrenia, Bipolar Disorder and Autism Spectrum Disorder, and that map to lncRNA genes that are dynamically expressed in the CNS (Lin et al., 2011). Together, the expression of lncRNAs in the brain are tightly regulated during neuronal development and aging. Changes in their expression levels during aging and in neurodegenerative diseases further imply that lncRNAs can play crucial roles in pathological events in the brain and underscore their functional importance in brain development and homeostasis.

## Mechanisms of lncRNAs in Neurological Disorders

Perhaps the most intriguing feature of lncRNAs is their association with disease. Emerging evidence emphasizes the importance of lncRNAs in CNS development and (dys)function. Genome-wide association studies and comparative transcriptomic analyses link lncRNA deregulation and dysfunction to multiple human diseases, including a wide range of neurodevelopmental, neuropsychiatric, and neurodegenerative conditions (Salta and De Strooper, 2017; Salvatori et al., 2020). The identification of multiple natural antisense transcripts (NATs), a class of lncRNAs, at distinct human loci associated with hereditary neurodegenerative disorders including Alzheimer’s Disease, Frontotemporal Dementia, Parkinson’s Disease, Amyotrophic Lateral Sclerosis and Huntington’s Disease further illustrates the potential role of lncRNAs in the expression regulation of neurodegeneration-related genes (Zucchelli et al., 2019).

**TABLE 1:** LncRNA regulatory mechanisms and their contribution to CNS disorders.

**Function**	**LncRNA**	**Binding partners**	**Mode of action**	**Associated disease**	**References**
**Epigenetic regulation**	*BDNF-AS*	PRC2	Act as scaffolds to recruit chromatin modifiers to *BDNF or SMN* promoter region, respectively, and transcriptionally repress their targets	AD, HD, MDD	Modarresi et al., 2012
	*SMN-AS1*	PRC2		SMA	d’Ydewalle et al., 2017; Woo et al., 2017
	*HAR1F/HAR1R*	REST	Mutant HTT re-locates REST to the nucleus and represses *HAR1* non-coding locus	HD	Johnson et al., 2010
	*TUG1, MEG3*	REST, PRC2	ND	HD	Johnson, 2012; Khalil et al., 2009; Myers et al., 2011;
	*DGCR5*	REST	ND	HD, SZ	Myers et al., 2011; Johnson, 2012; Meng et al., 2018
**Transcriptional regulation**	*UBE3A-ATS*	ND	Represses paternal copy of *UBE3A* (unclear whether due to the presence of the antisense lncRNA or due to locus transcription)	AS	Meng et al., 2012
	*LRP1-AS*	HMGB2	Acts as molecular decoy by binding to HMGB2 and inhibiting its ability to promote SREBP1a-dependent transcription of *LRP1*	AD	Yamanaka et al., 2015
	*FMR4*	ND	Proposed to negatively regulate *MBD4* transcription, a transcriptional repressor involved in DNA repair and apoptosis, via a *trans*-acting mechanism	FXS, FXTAS	Khalil et al., 2008; Peschansky et al., 2015
	*C9orf72*	Multiple RBPs	Sense and antisense transcripts accumulate in RNA foci and might function as molecular decoys for RBPs, including splicing factors, affecting their function	c9FTD/ALS	Lee et al., 2013; Barker et al., 2017
	*MIAT/Gomafu*	QKI, SRSF1	Acts as a splicing factor scaffold by binding to QK1 and SRSF1 and regulate alternative splicing of *DISC1* and *ERBB4* genes	SZ	Barry et al., 2014
	*51A/SORL1-AS*	*SORL1*	Downregulates canonical *SORL1* variant A through alternative splicing, increasing Aβ formation	AD	Ciarlo et al., 2013
	*17A*	*GPR51*	Shifts alternative splicing of *GPR51* toward an isoform that abolishes GABA B2 intracellular signaling	AD	Massone et al., 2011
**Post-transcriptional regulation**	*BACE1-AS*	*BACE1*	Positively regulates the levels of *BACE1* mRNA by masking binding site for miR-485-5p, promoting Aβ synthesis	AD	Faghihi et al., 2008, 2010;
	*SHANK2-AS*	*SHANK2*	Directly binds to *SHANK2* mRNA, decreasing its mRNA stability and expression	ASD, SZ	Luo et al., 2018
	*PINK1-AS*	*PINK1*	Directly binds to the mRNA of the *svPINK1* splice variant, stabilizing its expression by formation of an RNA duplex	PD	Scheele et al., 2007
	*HOTAIR*	*LRRK2*	Directly binds to *LRKK2* mRNA, stabilizing its expression by formation of an RNA duplex and inducing neuronal apoptosis	PD	Wang S. et al., 2017
	*HTT-AS*	*HTT*	Represses mutant *HTT* expression via a RISC-dependent mechanism	HD	Chung et al., 2011
	*AS UCHL1*	*UCHL1*	Makes use of a SINE B2 sequence to upregulate translation of *UCHL1*, therefore belonging to the SINEUP subclass of lncRNAs	AD, PD	Carrieri et al., 2012; Zucchelli et al., 2015
	*MAPT-AS1*	*MAPT*	Short elements within *MAPT-AS1* MIR sequence interfere with the binding of *MAPT* mRNA to ribosomes, thus blocking translation	AD, PD	Simone et al., 2021
Structural	MALAT1, NEAT1	Multiple (splicing	Structural components of	FTLD-TDP, PD	Tollervey et al., 2011;
function		factors, miRNAs,	nuclear speckles		Nishimoto et al., 2013;
		epigenetic regulators,	(MALAT1)	FTLD-TDP, ALS, HD, SZ	Sunwoo et al., 2017;
		transcription factors,	and paraspeckles (NEAT1);		Shelkovnikova et al., 2018;
		chromatin)	multiple roles in gene		Katsel et al., 2019;
			regulation at epigenetic,		Xia et al., 2019
			transcriptional and		
			post-transcriptional levels in		
			a context-dependent		
			manner		

**BDNF-AS*, brain-derived neurotrophic factor-antisense; *SMN-AS1*, survival motor neuron-antisense 1; *HAR1F*, human accelerated region 1 forward; *HAR1*, human accelerated region 1 reverse (F-forward; R-reverse); *TUG1*, taurine upregulated gene 1; *MEG3*, maternally expressed 3; *DGCR5*, DiGeorge syndrome critical region gene 5 *UBE3A-ATS*, ubiquitin ligase E3A-antisense, *LRP1-AS*, low-density lipoprotein receptor-related protein 1-antisense; *FMR4*, fragile X mental retardation 4; *c9orf72*, chromosome 9 open reading frame 72; *MIAT*, myocardial infarction associated transcript; *SORL1-AS*, sortilin related receptor 1-antisense; *BACE1-AS*, β-site APP cleaving enzyme 1-antisense; *SHANK2-AS*, SH3 and multiple ankyrin repeat domains 2-antisense; *PINK1-AS*, PTEN-induced kinase 1-antisense; *HOTAIR*, HOX transcript antisense intergenic RNA; *HTT-AS*, huntingtin-antisense; *AS UCHL1*, antisense to ubiquitin carboxy-terminal hydrolase L1; *MAPT-AS1*, microtubule associated protein transcript antisense 1; *MALAT1*, metastasis-associated lung adenocarcinoma transcript 1; *NEAT1*, nuclear paraspeckle assembly transcript 1; PRC2, polycomb repressive complex 2; REST, RE1-silencing transcription factor; ND, not defined; RBPs, RNA-binding proteins; HMGB2, high mobility group Box 2; QKI, quaking homolog, KH domain RNA binding; SRSF1, serine and arginine rich splicing factor 1; *SORL1*, sortilin related receptor 1; *BACE1-AS*, β-site APP cleaving enzyme 1; *SHANK2*, SH3 and multiple ankyrin repeat domains 2; *PINK1*, PTEN-induced kinase 1, *GPR51*, G protein-coupled receptor 51; *LRRK2*, leucine-rich repeat kinase 2; SREBP1a, sterol regulatory element binding protein 1a; *MBD4*, methyl-CpG binding domain 4; *DISC1*, disrupted-in-schizophrenia 1; *ERBB4*, Erb-B2 receptor tyrosine kinase 4; SINEUP, SINE B2 sequence to UP-regulate translation; MIR, mammalian-wide interspersed repeat; AD, Alzheimer’s disease; HD, Huntington’s disease; MDD, major depressive disorder; SMA, spinal muscular atropy; SZ, schizophrenia; AS, Angelman Syndrome; FXS, Fragile X Syndrome; FXTAS, Fragile X-associated Tremor/Ataxia Syndrome; c9FTD/ALS, c9orf72-associated Frontotemporal Dementia and Amyotrophic Lateral Sclerosis (ALS); ASD, autism spectrum disorders; PD, Parkinson’s disease; FTLD-TDP, frontotemporal lobar degeneration associated with TDP-43.*

**FIGURE 3:**
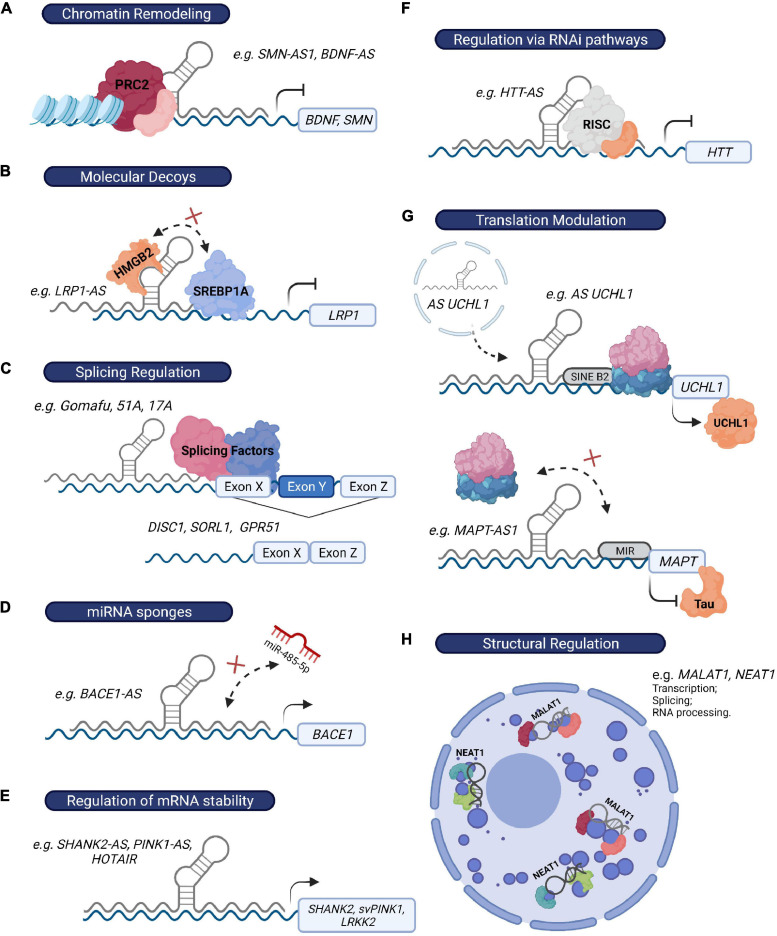
LncRNAs regulate gene expression through multiple mechanisms. **(A)** LncRNAs can recruit chromatin modifiers (e.g., PRC2 complex) to their target-gene promoters and epigenetically influence their expression (e.g., *BDNF-AS* and *SMN-AS1*). **(B)** Alternatively, they can modulate transcription by acting as molecular decoys and sequester specific chromatin and non-chromatin modulators, transcription factors, or other regulatory proteins from the promoters of their target genes (e.g., *LRP1-AS*). **(C)** LncRNAs can also influence transcription by affecting the alternative splicing of their target genes (e.g., *Gomafu, 51A, 17A*). **(D)** Some lncRNAs regulate mRNA turnover by acting as miRNA “sponges” (e.g., *BACE1-AS*), as they prevent miRNAs from binding their target genes. **(E)** Additionally, they can directly bind to their target mRNAs, leading to the formation of an RNA-RNA duplex and therefore influencing mRNA stability (*SHANK2-AS*, *PINK1-AS*, and *HOTAIR*). **(F)** LncRNAs can also exert their function via recruitment of the RISC complex to their target mRNA (e.g., *HTT-AS*). **(G)** Some lncRNAs directly modulate translation efficiency of their target genes through the presence of specific sequences that either promote (e.g., SINE B2 element in *AS UCHL1*) or prevent (e.g., MIR sequence in *MAPT-AS1*) the association of ribosome machinery with their target mRNAs. **(H)** Finally, *NEAT1* and *MALAT1* exert a crucial structural function in the formation of paraspeckles and speckles, respectively, two nuclear structures that regulate multiple mechanisms, including RNA transcription, splicing and processing.

However, the association of altered expression of specific lncRNAs in brain disorders is not hard proof of biological relevance, and functional studies investigating the role of these lncRNAs are still needed. Since lncRNA function is intrinsically influenced by their subcellular localization (Chen, 2016; Bridges et al., 2021), we will separately discuss lncRNAs that exert their function at the epigenetic or transcriptional level in the nucleus, and those responsible for post-transcriptional regulatory mechanisms in the cytoplasm (Table 1 and Figure 3). Nevertheless, some lncRNAs might be present in both compartments and exert different functions depending on their location (Bridges et al., 2021).

### Epigenetic Regulation

Numerous nuclear lncRNAs with potential roles in neurological disorders associate with chromatin where they interact with a wide variety of proteins to enhance or repress their binding and activity at specific DNA regions (Guttman et al., 2011; Roberts et al., 2014; Yao et al., 2019). Specifically, lncRNAs can recruit chromatin modifiers to their target-gene promoters and activate or inhibit their transcription in *cis* (close to their transcription sites) or in *trans* (regulation is exerted at distant loci). Alternatively, they can act as molecular decoys, sequestering specific chromatin modulators from the promoters of target genes. Finally, they can directly interact with DNA and generate DNA-RNA hybrid structures, such as R-loops, which ultimately influence chromatin accessibility and remodeling (Yao et al., 2019; Statello et al., 2021).

#### LncRNAs Interact With the PRC2 Complex

Many lncRNAs interact with the PRC2 complex, constituted by a group of polycomb proteins that mediate epigenetic silencing in diverse biological processes (Davidovich and Cech, 2015; Balas et al., 2021). The NAT *BDNF*-*AS* is transcribed antisense to the *BDNF* gene, which encodes a neurotrophic factor essential for neuronal development and maintenance (Modarresi et al., 2012; Miranda et al., 2019). Moreover, BDNF protein levels are reduced in various neurodegenerative disorders including Alzheimer’s Disease (Laske et al., 2011), Huntington’s Disease (Ferrer et al., 2000; Zuccato and Cattaneo, 2007), and Major Depressive Disorder (Molendijk et al., 2011; Emon et al., 2020). *BDNF-AS* knockdown leads to an increase in *BDNF* mRNA and protein levels both *in vitro* and *in vivo* by reducing the recruitment of the histone lysine methyltransferase EZH2, a component of PRC2, and consequently decreasing the levels of the repressive chromatin mark in the *BDNF* promoter region. Even though it remains unclear how *BDNF-AS* recruits the PRC2 components to the *BDNF* locus, this interaction could be of relevance in disorders where an upregulation of BDNF protein levels in the brain is needed (Modarresi et al., 2012).

Two independent studies identified *SMN-AS1* as an antisense lncRNA that arises from the *SMN* locus and recruits the PRC2 complex to transcriptionally repress *SMN* expression and to have relevant implications for Spinal Muscular Atrophy (SMA) (d’Ydewalle et al., 2017; Woo et al., 2017). SMA is a genetic disorder caused by an autosomal recessive mutation of deletion of the *SMN1* gene which results in atrophy of skeletal muscles due to a progressive motor neuron loss. Increasing the expression of the homologous *SMN2* gene has been shown to functionally compensate for the loss of *SMN1* and ameliorate disease severity by upregulating SMN protein levels (d’Ydewalle and Sumner, 2015). Knockdown of *SMN-AS1* with antisense oligonucleotides (ASOs) can dissociate PRC2 from the *SMN* promoter and increase SMN expression in both *in vitro* and *in vivo* SMA models (d’Ydewalle et al., 2017; Woo et al., 2017). Combining *SMN2* splice-switching oligonucleotides and ASOs targeting *SMN-AS1* further boosted the levels of SMN protein and improved survival of SMA mice compared to either therapy by itself. These findings suggest that targeting this lncRNA combined with the FDA approved *SMN2* splice-switching oligonucleotides (*Spinraza*) could represent a valuable therapeutic approach for severe SMA cases characterized by very low SMN levels (U.S. Food & Drug Administration, 2016; d’Ydewalle et al., 2017).

#### LncRNAs Interact With Other Chromatin Modifiers

Chromatin-remodeling complexes other than PRC2 might be involved in neurological disorders through their interaction with lncRNAs. Huntington’s Disease (HD) is a polyglutamine (PolyQ)-related disorder caused by an expanded CAG repeat (>36) in the first exon of the *HTT* gene. The repeat expansion results in the production of a mutant neurotoxic form of the HTT protein that triggers a progressive degeneration of cortical and striatal neurons. Although the mechanisms behind neuronal loss in HD are not yet completely understood, aberrant chromatin remodeling and transcriptional dysregulation seem to represent key features in the pathogenesis (Benn et al., 2008). For instance, during HD pathology, the transcriptional repressor REST is abnormally relocated to the nucleus by a mutant HTT-dependent mechanism, resulting in the repression of many REST target genes (Zuccato et al., 2007). The *HAR1* non-coding locus is directly targeted by REST, which might explain the reduction in *HAR1F* and *HAR1R* transcript levels observed in the striatum of HD patients (Johnson et al., 2010). The expression of other lncRNAs with potential epigenetic regulatory functions is dysregulated in the brains of HD patients (Johnson, 2012). These include the putative REST targets *DGCR5*, *NEAT1*, and *MEG3* (Myers et al., 2011), and *TUG1* (Johnson, 2012). Interestingly, *TUG1* and *MEG3* can interact with PRC2 (Khalil et al., 2009). Therefore, it is likely that altered levels of some of these lncRNAs might influence gene expression patterns during disease. Similarly, *DGCR5* has recently also been proposed to regulate the expression of genes associated with Schizophrenia (Meng et al., 2018).

### Transcriptional Regulation

Multiple lncRNAs regulate their target genes at the transcriptional level. Importantly, two mechanisms may play a role in this regulation: the lncRNA transcript can influence the transcription of neighboring loci, and/or the act of transcription of the lncRNA itself can drive chromatin remodeling and affect the expression of other genes (Statello et al., 2021).

#### Is It the Transcript or Transcription?

*UBE3A-ATS* is a nuclear lncRNA that has been implicated in Angelman syndrome (AS), a severe neurodevelopmental disorder caused by a maternal deficiency of the imprinted gene *UBE3A* (Meng et al., 2012). Although patients carry at least one functional copy of the paternal *UBE3A*, in neurons this allele is silenced by the antisense lncRNA. Whether silencing of *UBE3A* by *UBE3A-ATS* occurs due to the presence of the antisense lncRNA or due to transcription of this locus is unclear (Meng et al., 2012). Nonetheless, reducing *UBE3A-ATS* levels to restore UBE3A protein levels has been proposed as a potential therapeutic intervention for AS (Meng et al., 2013, 2015; Wolter et al., 2020).

#### LncRNAs Act as Molecular Decoys

LncRNAs can also act as molecular decoys for other RNA-binding proteins (RBPs) including transcription and splicing factors. For example, Yamanaka et al. (2015) identified a NAT associated with the *LRP1* gene, designated as *LRP1*-AS (*Lrp1-AS* in mouse), which has been implicated in Alzheimer’s Disease (AD) pathology. AD is the main cause of dementia worldwide and two hallmarks of this neurodegenerative disorder are the accumulation of extracellular amyloid-β (Aβ)-containing plaques and intracellular neurofibrillary tangles composed of hyperphosphorylated and aggregated Tau protein within the patients’ brains (Scheltens et al., 2021). LRP1 protein plays a major role in different aspects of AD (Tachibana et al., 2019; Rauch et al., 2020). Modulation of *Lrp1-AS* levels in a mouse cell line revealed that this lncRNA negatively regulates *Lrp1* expression both at the mRNA and protein levels. *Lrp1-AS* directly binds to HMGB2, a non-histone chromatin modifier, inhibiting its ability to promote SREBP1A-dependent transcription of *Lrp1*. Accordingly, increased levels of *LRP1-*AS and a reduction in *LRP1* expression in human AD brain samples compared to age-matched controls were observed (Yamanaka et al., 2015). However, further studies are needed to specifically investigate the role of *LRP1*-*AS* in the transcriptional regulation of *LRP1* in the human brain and its functional effects in AD pathogenesis.

Development of Fragile X Syndrome (FXS) or the related disease Fragile X-associated Tremor/Ataxia Syndrome (FXTAS) is caused by the presence of a triple CGG repeat motif in the 5′ untranslated region (5′-UTR) of the *FMR1* gene (Khalil et al., 2008; Peschansky et al., 2015; Huang et al., 2019). Unaffected individuals typically carry 5–54 repeats while 55–200 repeats are considered a premutation leading to the development of FXTAS. A full mutation occurs when > 200 repeats are present, and this is necessary to develop FXS. In most FXS patients, *FMR1* is hypermethylated and transcriptionally silenced resulting in decreased *FMR1* mRNA and FMRP protein levels, impacting neurogenesis (Sunamura et al., 2018). Intriguingly, the *FMR1* locus is particularly complex and encodes several lncRNAs. The expression of these lncRNAs are differentially affected by the repeat expansion mutations suggesting that they could potentially explain the distinct clinical features of FXS and FXTAS (Ladd et al., 2007; Khalil et al., 2008; Pastori et al., 2014; Vittal et al., 2018; Huang et al., 2019). One of these lncRNAs, *FMR4*, overlaps with the repeat region and is silenced in FXS patients but upregulated in premutation carriers (Khalil et al., 2008; Peschansky et al., 2015). Previously, Khalil et al. (2008) have shown that even though *FMR4* does not regulate *FMR1* expression, it exhibits anti-apoptotic functions in human cell lines. Later on, and consistent with a role at the transcriptional level, *FMR4* was found to be primarily associated with chromatin, and a discordant expression between the lncRNA and *MBD4*, a transcriptional repressor involved in DNA repair mechanisms and regulation of apoptosis (Bellacosa, 2001), was observed during human neuronal precursor cells development. This suggests a *trans*-acting regulatory role for *FMR4* independent of *FMR1* which might be impaired in Fragile X repeat expansion-associated diseases (Peschansky et al., 2015).

The presence of hexanucleotide GGGGCC (G4C2) repeat expansions in the *C9orf72* gene represents the most common genetic cause of Frontotemporal Dementia (FTD) and Amyotrophic Lateral Sclerosis (ALS), two devastating neurodegenerative disorders commonly designated as c9FTD/ALS (Barker et al., 2017). While the mechanisms by which these repeat expansions lead to c9FTD/ALS are not fully understood, both sense and antisense RNA foci comprising *C9orf72* RNA are widely distributed across the CNS of these patients (Gendron et al., 2013; Zu et al., 2013; Cooper-Knock et al., 2015). A potential mechanism by which RNA foci are thought to cause neurotoxicity is by directly sequestering specific RBPs and disrupting their function (Barker et al., 2017). Ultimately, this leads to a wide range of RNA misprocessing events, including aberrant alternative splicing. In support of this, Lee et al. (2013) transfected human neuroblastoma cells with different G4C2 repeat lengths and revealed that longer repeat lengths originate RNA foci that co-localize with a subset of RBPs involved in alternative splicing, which include SF2, SC35 and hnRNP-H. Furthermore, hnRNP-H directly binds to G4C2 repeat sequences and associate with RNA foci in both transfected cells and in the brains of c9FTD/ALS patients (Lee et al., 2013).

#### LncRNAs Influence Alternative Splicing

Another mechanism by which lncRNAs can influence transcription is by affecting the alternative splicing of their target genes (Yao et al., 2019). Schizophrenia is a complex mental disorder that affects about 1% of the population, and it is likely to result from different genetic, epigenetic and environmental factors that culminate in neurodevelopmental abnormalities and brain dysfunction (Morikawa and Manabe, 2010; Barry et al., 2014). Many genes associated with Schizophrenia are aberrantly spliced, including *DISC1* (Nakata et al., 2009) and *ERBB4* (Law et al., 2007). In line with this, *Gomafu* can act as a splicing factor scaffold, directly binding to the splicing factors QKI and SRSF1 to regulate alternative splicing of *DISC1* and *ERBB4* in human neurons derived from induced pluripotent stem cells (Barry et al., 2014). Notably, abnormal alternative splicing patterns upon ASO-mediated knockdown of *Gomafu in vitro* match those observed for these two genes in post-mortem brains from individuals affected by Schizophrenia. Finally, *Gomafu* levels are reduced in cortical samples from patients compared to controls further suggesting a role for *Gomafu* in Schizophrenia pathology (Barry et al., 2014).

In AD, the lncRNAs *51A*/*SORL1*-AS and *17A* have both been suggested to interfere with the alternative splicing of their neighboring genes - *SORL1* encoding Sortilin-1 and *GPR51* encoding GABA receptor B2, respectively - and their expression is upregulated in the brain tissue from AD patients (Massone et al., 2011; Ciarlo et al., 2013).

### Post-transcriptional Regulation

LncRNAs can be exported to the cytoplasm, where they regulate gene expression at the post-transcriptional level by influencing mRNA turnover, modulating translation or by interfering with post-translational modifications (Yao et al., 2019; Salvatori et al., 2020).

#### LncRNAs “Sponge” miRNAs

Some lncRNAs regulate mRNA turnover of their target genes by competing with *miRNA* binding sites by acting as *miRNA* “sponges” (Su et al., 2018; Yao et al., 2019). For instance, *BACE1-AS* has been identified as an antisense lncRNA associated with the *BACE1* gene (Faghihi et al., 2008). *BACE1-AS* regulates the levels of *BACE1* mRNA by masking *BACE1* mRNA binding site for miR-485-5p (Faghihi et al., 2010). *BACE1* encodes a secretase involved in the biosynthesis of Aβ and plays a central role in the amyloid cascade in AD pathophysiology (Hampel et al., 2020). In the presence of cell stressors including Aβ_1__–__42_, *BACE1-AS* levels increase thereby raising the levels of both *BACE1* mRNA and protein. This in turn stimulates the production of Aβ_1__–__42_, at least *in vitro*, which may lead to a detrimental accumulation of toxic Aβ aggregates (Faghihi et al., 2008). Moreover, different brain regions from AD patients exhibit increased levels of this lncRNA compared to control subjects indicating a direct role for *BACE1-AS* in driving AD pathology (Faghihi et al., 2008).

#### LncRNAs Regulate mRNA Stability

Another possibility is that lncRNA transcripts directly bind to their target mRNA, originating an RNA duplex and affecting mRNA stability by either recruiting proteins that promote mRNA degradation or, on the contrary, by acting as molecular decoys for RBPs involved in mRNA decay (Yao et al., 2019). Autism Spectrum Disorder (ASD) comprises a range of heterogeneous neurodevelopmental disorders characterized by cognitive, social and sensory impairments (Lord et al., 2020). Many are co-expressed with ASD risk genes in the developing brain (Cogill et al., 2018). For instance, the expression *SHANK2*-*AS*, a lncRNA transcribed antisense to the *SHANK2* gene, is upregulated and its levels negatively correlate with *SHANK2* mRNA and protein levels in ASD patients compared to control individuals (Wang et al., 2015; Luo et al., 2018). Mutations in the *SHANK2* gene, which encodes a post-synaptic density scaffold protein, have been identified as risk factors for ASD (Sato et al., 2012; Zaslavsky et al., 2019) and Schizophrenia (Peykov et al., 2015). *SHANK2*-*AS* can directly bind to *SHANK2* mRNA, consequently decreasing its expression *in vitro* (Luo et al., 2018). Furthermore, overexpression of *SHANK2*-*AS* or downregulation of *SHANK2* reduces neurite length and number in a human neuronal cell line suggesting that abnormal expression of *SHANK2*-AS may affect neuronal structure and growth by downregulating *SHANK2* expression, and thus directly contribute to ASD pathology (Luo et al., 2018).

Parkinson’s Disease (PD) is a neurodegenerative disorder generally characterized by the accumulation of α-synuclein (encoded by the *SNCA* gene) aggregates within Lewy bodies or Lewy neurites and degeneration of dopaminergic neurons, particularly in the substantia nigra. While most PD cases are sporadic, familial forms of the disease can occur and result from mutations in a group of genes that include *LRRK2*, *PARK2*, *PARK7*, *PINK1* or the *SNCA* gene itself (Bandres-Ciga et al., 2020). The antisense lncRNA *PINK1-AS* stabilizes the expression of a *PINK1* splice variant (svPINK1) *in vivo* through the formation of an RNA duplex (Scheele et al., 2007). This interaction might have important implications in multiple disorders besides PD (Wilhelmus et al., 2011), as *PINK1* is thought to have a neuroprotective role against stress-induced mitochondrial dysfunction, oxidative stress and apoptosis (Deas et al., 2009). In addition, the lncRNA *HOTAIR* has also been implicated in PD pathology by increasing *LRKK2* mRNA stability and inducing dopaminergic neuronal apoptosis in a human neuroblastoma cell line (Wang S. et al., 2017). There are also several lncRNAs identified that can arise from the antisense strand in the *SNCA* locus either overlapping with the SNCA gene in the 5′ or 3′end (Zucchelli et al., 2019). Although their role in regulating *SNCA* expression is still enigmatic, expression analysis confirmed that some of these lncRNAs are co-expressed with *SNCA*, including in the substantia nigra (Zucchelli et al., 2019).

#### LncRNAs Act via the RNAi Pathway

The lncRNA *HTT-AS* originates from the HD repeat locus containing the repeat region and represses mutant HTT expression via an RNA-induced silencing complex (RISC)-dependent mechanism (Chung et al., 2011). In human cells, overexpression of the *HTT-AS* transcript reduces *HTT* mRNA levels, while knocking down the lncRNA upregulates *HTT* transcripts. Interestingly, reduced levels of *HTT-AS* were observed in the frontal cortex from HD patients compared to control individuals suggesting a potential protective role in HD in which the presence of expanded repeats reduces *HTT-AS* expression, removing its inhibitory effect on *HTT* expression (Chung et al., 2011).

#### LncRNAs Modulate Translation

Finally, some lncRNAs directly modulate translation efficiency of genes associated with neurological disorders (Muddashetty et al., 2002; Salta and De Strooper, 2017). Carrieri et al. (2012) have identified *AS Uchl1* as an antisense lncRNA associated with the *Uchl1* gene. UCHL1 is a highly abundant neuronal protein involved in neuronal development (Reinicke et al., 2019), dopaminergic neuron differentiation (Carrieri et al., 2015) and regulation of the ubiquitin-proteasome pathway (Yichin et al., 2002). *UCHL1* gene variants are associated with susceptibility to PD (Ying et al., 2015), and oxidative modifications and downregulation of UCHL1 protein are observed in sporadic cases of both AD and PD patients (Choi et al., 2004). *AS Uchl1* shuttles from the nucleus to the cytoplasm upon rapamycin induced mTOR activity in a dopaminergic cellular model, where it targets *Uchl1* mRNA to active polysomes to facilitate its translation and upregulate Uchl1 protein levels (Carrieri et al., 2012). Owing to the presence of an embedded repetitive sequence SINE B2 in the inverted orientation at the non-overlapping part of the transcript, *AS Uchl1* has been considered the representative member of a recently identified functional NAT class designated as SINEUPs. These molecules require a SINE B2 sequence to UP-regulate translation of their target mRNA. Importantly, due to their ability to increase the translation of virtually any gene of interest, the development of synthetic SINEUPs that target the antisense sequence of the target mRNA might constitute an interesting therapeutic approach to enhance protein synthesis (Zucchelli et al., 2015).

The very recent identification of NATs with embedded mammalian-wide interspersed repeat (MIR) sequences – referred to as MIR-NATs – associated with protein-coding genes tied to neurodegenerative disorders has revealed a new class of lncRNAs that mediate gene regulation at the translational level through a MIR-dependent mechanism (Simone et al., 2021). One of these MIR-NATs, *MAPT-AS1*, is associated with the *MAPT* gene – encoding the Tau protein - and has been suggested to repress Tau translation by competing for ribosomal RNA pairing with the *MAPT* mRNA internal ribosome entry site (Simone et al., 2021).

#### The Nucleus Takes It All

*NEAT1* and *MALAT1* (also known as *NEAT2*) are two human lncRNAs conserved within the mammalian lineage which regulate key nuclear functions (Hutchinson et al., 2007; Zhang et al., 2017; An et al., 2018). Despite displaying an adjacent genomic location, these two RNAs exert their function in related but distinct nuclear subdomains (West et al., 2014). *MALAT1* is localized in nuclear speckles which are enriched in serine- and arginine-rich (SR) splicing factors, while *NEAT1* is a structural component of nuclear bodies called paraspeckles, also composed by multiple proteins with reported roles in transcription and RNA processing (West et al., 2014). Therefore, both lncRNAs play important regulatory roles in many cellular pathways that are commonly affected in neurological diseases (Zhang et al., 2017; An et al., 2018). The expression of both *MALAT1* and *NEAT1* is markedly upregulated in the brains from Frontotemporal Lobar Degeneration associated with TDP-43 (FTLD-TDP) patients (Tollervey et al., 2011). Moreover, this differential expression can explain an increased binding of TDP-43 protein to these lncRNAs in FTLD-TDP patient samples, supporting a role for lncRNAs and TDP-43 in the regulation of splicing in the brain with direct implications for neurodegenerative diseases (Tollervey et al., 2011).

Although a large proportion of the genetic risk for familial ALS (fALS) is still elusive, mutations in *SOD1*, *TARDBP* (TDP-43), *FUS* and *C9orf72* genes are linked to the onset of fALS (Mejzini et al., 2019). Both TDP-43 and FUS/TLS are enriched in paraspeckles in cultured cells where they directly bind *NEAT1_2* transcript. Analysis of human spinal motor neurons revealed upregulated levels of *NEAT1_2*, and enhanced paraspeckle formation was confirmed in two independent cohorts of ALS cases compared to controls (Nishimoto et al., 2013; Shelkovnikova et al., 2018). The formation of paraspeckles has been linked to the loss of TDP-43 function in cells, suggesting a protective role for these structures (Shelkovnikova et al., 2018).

A microarray analysis demonstrated upregulated levels of *NEAT1* in human HD postmortem brain patients and in a mouse model of HD (Sunwoo et al., 2017). Transfection of *NEAT1* in a mouse cell line increases cell viability under oxidative stress, an observation that aligns with a previous report describing the involvement of *NEAT1* in cell survival pathways under stress conditions (Choudhry et al., 2015). Thus, these studies support the idea of a protective role for this lncRNA in non-physiological settings (Sunwoo et al., 2017). Conversely, expression of *NEAT1* is reduced in cortical brain regions from Schizophrenia patients. Furthermore, RNA-seq analysis performed in the frontal cortex of *NEAT1* deficient mice indicated that pathways related to oligodendrocytes differentiation and RNA post-translational modifications are significantly impacted (Katsel et al., 2019). These results are in line with previously discussed data indicating that *NEAT1* expression is dramatically changed during mouse oligodendrocyte-lineage specification (Mercer et al., 2010). Additionally, *NEAT1*^–/–^ mice displayed a significant reduction in the numbers of oligodendrocyte-lineage cells and impaired expression of genes related to myelination, supporting a role for *NEAT1* in oligodendrocyte function and related abnormalities in Schizophrenia pathology (Haroutunian et al., 2014; Katsel et al., 2019).

Finally, *MALAT1* was found to bind to α-synuclein protein and increase its stability in a human neuroblastoma cell line (Zhang et al., 2016). Accordingly, inhibition of *MALAT1* using resveratrol was found to increase miR-129 levels, consequently downregulating *SNCA* expression and improving disease-related phenotypes in a PD mouse model (Xia et al., 2019).

## Targeting lncRNAs in Neurological Disorders: Trash or Treasure?

Around 30 years ago, gene transcription was perceived as a process mostly regulated by protein transcription factors and RNA was largely seen as an intermediary between DNA and protein; RNA had merely a supportive role in the translation of genetic information into diverse functional programs within the cells. However, the recent identification of multiple endogenous RNA classes with unexpected functions in gene regulation, including lncRNAs, has raised the interest to exploit RNA-based therapies (Wahlestedt, 2013; Roovers et al., 2018). To date, the biological functions of many annotated lncRNAs remain largely unexplored. The high unmet need for efficacious disease-modifying therapies for many of the neurodegenerative diseases mentioned in this review underscores the necessity to take bold steps and invest more in lncRNA research in order to open up innovative therapeutic opportunities for disorders of the brain.

### Targeting LncRNAs at the DNA Level

**FIGURE 4:**
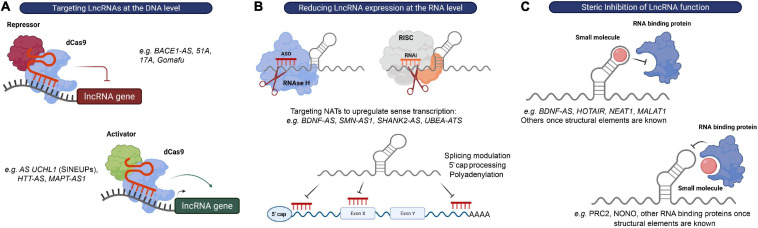
Strategies to target lncRNAs. **(A)** DNA editing: CRISPRi and CRISPRa tools can be used to transcriptionally silence or activate lncRNA expressing loci, respectively. **(B)** Modulation of RNA levels: ASOs and siRNAs can be used to reduce lncRNA levels in order to alter the expression of their associated protein-coding genes. recruits RBPs that mediate RNA processing events, such as 5′ capping, splicing or polyadenylation, to modulate expression or RNAse H to promote degradation of the lncRNA transcript, respectively, while RNAi induces RISC-mediated cleavage of the lncRNA transcript. **(C)** Steric inhibition: small molecules target secondary and tertiary structures of lncRNAs and/or their binding partners to block their interaction.

Recent progress in genome-editing techniques, such as CRISPR-based methods including CRISPR-interference (CRISPRi) and CRISPR-activation (CRISPRa), has brought the exciting possibility of transcriptionally silence or activate lncRNA expressing loci, and clearly demonstrate that these tools will be crucial for a better understanding of lncRNA biology (Jinek et al., 2012; Cong et al., 2013; Mali et al., 2013; Qi et al., 2013; Gilbert et al., 2014; Thakore et al., 2015; Zhu et al., 2016; Abudayyeh et al., 2017; Liu S. J. et al., 2017; Chen et al., 2019; Phelan and Staudt, 2020; Wolter et al., 2020; Xu D. et al., 2020; Zhang et al., 2021). One way to achieve such transcriptional modulation is to use a dead-Cas9 approach. In this approach, a mutant form of Cas9 without endonuclease activity is fused to transcriptional repressors or activators to achieve transcriptional silencing or activation, respectively, of a specific gene promoter (Liu S. J. et al., 2017; Arun et al., 2018) (Figure 4A). Furthermore, Cas9-based gene therapy was shown to successfully reduce the expression of *UBE3A-ATS* and activate the paternal *UBE3A* in a mouse model of AS. Early treatment with an adeno-associated viral (AAV) delivery system designed to activate the expression of paternal *UBE3A* for at least 17 months ameliorated disease phenotype in AS mice and provided proof of concept that this approach is therapeutically relevant (Wolter et al., 2020). Onasemnogene abeparvovec, an AAV-based therapy carrying a functional copy of the *SMN* gene, was approved in May 2019 as the first gene therapy for SMA in the United States and illustrates that AAV approaches can lead to clinical successes for devastating neurological diseases (Hoy, 2019). While recent advances in CRISPR-based techniques demonstrate great potential for the discovery of disease mechanisms and identification of new therapeutic targets, there are still some challenges and risks that need to be considered prior to their use as therapy for neurological disorders. These include finding an effective CNS delivery method, the irreversibility of DNA editing, and safety concerns related to undesired on-target and off-target effects (Sun and Roy, 2021). For instance, a genome-wide association analysis found that almost two-thirds of lncRNA loci are at risk of inadvertently influencing the expression of neighboring genes upon CRISPR-mediated targeting (Goyal et al., 2017). Thus, future research will be crucial to explore the full potential of these modalities as therapeutics.

### Reducing LncRNA Expression at the RNA Level

The possibility of modulating RNA expression with oligonucleotides provides a relatively straightforward strategy to impact virtually any RNA of interest and to target previously “undruggable” portions of our genome (Arun et al., 2018). At the moment, there are two major strategies employing oligonucleotide-based therapeutics: ASOs and RNA-mediated interference (RNAi), which share the fundamental principle of exerting their catalytic activity by binding their target RNA through Watson-Crick base pairing (Watts and Corey, 2012). ASOs are single stranded nucleotide sequences that bind RNA primary sequences to either affect RNA processing events, such as 5′-cap formation, splicing and polyadenylation, induce RNA degradation via the recruitment of Ribonuclease H (RNase H), or repress translation (in the case of protein-coding genes) (DeVos and Miller, 2013). Alternatively, a complementary hybrid RNA strand will engage with the target RNA in the RISC complex to initiate its degradation in the RNAi mechanism (Hannon and Rossi, 2004) (Figure 4B).

Remarkably, both ASO- and siRNA-based strategies have been proven successful in reducing the expression of several lncRNAs with potential roles in neurological disorders (Scheele et al., 2007; Chung et al., 2011; Carrieri et al., 2012; Modarresi et al., 2012; Barry et al., 2014; Meng et al., 2015; Peschansky et al., 2015; Yamanaka et al., 2015; d’Ydewalle et al., 2017; Wang X. et al., 2017; Woo et al., 2017; Luo et al., 2018). However, their efficiency is at least partially dependent on the subcellular compartment in which the target lncRNA localizes (Lennox and Behlke, 2016). Specifically, while lncRNAs primarily located within the nucleus were shown to be easier to target using ASOs, the expression of cytoplasmic lncRNAs was more efficiently reduced using RNAi methods. Additionally, both strategies were able to suppress the levels of lncRNAs residing in both compartments, although ASOs performed overall better in this case (Lennox and Behlke, 2016). In line with these observations, RNAse H1-dependent ASOs robustly exert their function in both the nucleus and the cytoplasm (Liang et al., 2017). These studies highlight the importance of better understanding how and where lncRNAs play their functional roles to select the most appropriate therapeutic approach to target these molecules.

A particular attractive class of candidate targets for these approaches is the previously mentioned NATs. Most of these NATs affect the transcription and/or translation of their neighboring genes. Furthermore, many of these antisense lncRNAs have been shown to act as repressors of sense coding genes in a locus-specific manner (e.g., *SMN-AS1, BDNF-AS, SHANK2-AS*, and *UBE3A-ATS*). Since there is still a considerable lack of therapeutic approaches to upregulate gene expression, inhibition of these transcripts with siRNAs or ASOs (called “antagoNATs”) might be of particular interest to increase the expression of genes found downregulated in CNS disorders (Wahlestedt, 2013) (Figure 4B).

The recent approval of several ASOs and siRNAs for clinical intervention clearly demonstrates the huge therapeutic potential of RNA-based therapies for a wide range of human diseases, including HD, ALS, AD and FTD (Roberts et al., 2020). However, two main limitations need to be considered. First, systemic delivery to the CNS remains a limitation since oligonucleotides are unable to cross the blood-brain barrier (Roberts et al., 2020). Additionally, due to the presence of complex secondary and tertiary structures, their incorporation into large protein complexes, or their specific intracellular location, some lncRNAs might be hard to target using these tools (Gutschner et al., 2011; Wilusz et al., 2012; Brown et al., 2014; Pegueroles and Gabaldón, 2016).

### Steric Inhibition of LncRNA Function

Interfering with lncRNA function(s) rather than modulating its expression levels may represent an alternative therapeutic approach. An increasing body of evidence suggests that the function of many lncRNAs is largely mediated by their interaction with RBPs or protein complexes. Thus, a promising therapeutic option for targeting lncRNAs is the use of steric hindering antisense oligonucleotides or small molecules that affect these interactions by either (1) blocking the RNA-binding domain of the protein, or (2) directly binding to and disrupting secondary and tertiary structures in the lncRNA molecule (Meyer et al., 2020) (Figure 4C).

The variety of lncRNA binding partners and their unique structural features offer exceptional opportunities to target these RNAs using small molecules. Importantly, the development of new RNA sequencing techniques and structure determination assays such as SHAPE (Wilkinson et al., 2006), SHAPE-MaP (Smola et al., 2015), PARIS (Lu et al., 2016), or CROSSalign (Ponti et al., 2018) have made it possible to map the secondary and tertiary structures of lncRNAs. In addition, a database tool called LNCmap has been recently developed to explore correlations among diseases, small molecules and lncRNA signatures (Yang et al., 2017). Indeed, structural domains of several lncRNAs that interact with proteins or protein complexes, continue to be revealed (Smith et al., 2013; Mondal et al., 2015; Somarowthu et al., 2015; Liu F. et al., 2017; McCown et al., 2019; Balas et al., 2021).

*MALAT1* has been proposed to contain a bipartite triple helix at the 3′ end and single-point mutations that destabilize this structure have been shown to reduce *MALAT1* levels in cells, implying a crucial role for this structure in enabling *MALAT1* expression (Brown et al., 2014, 2016). The secondary structure of the inverted SINE B2 element embedded in the mouse *AS Uchl1* has also been recently revealed by Podbevšek et al. (2018). The authors found that removal of a structural motif containing a short hairpin abolishes the ability of *AS Uchl1* to upregulate UCHL1 protein levels, highlighting the importance of specific structural determinants of the SINE B2 sequence in the functionality of *AS Uchl1* (Podbevšek et al., 2018). Furthermore, the RBP NONO is an important component of paraspeckles and was shown to be recruited to these structures by specifically binding to highly abundant and conserved G-quadruplex motifs in the lncRNA *NEAT1* (Simko et al., 2020). These motifs represent the main structural element recognized by the catalytic subunit of PRC2 (Wang X. et al., 2017), which we already discussed as an important binding partner for many lncRNAs, including *HOTAIR* (Rinn et al., 2007), *XIST* (Bousard et al., 2019), *SMN-AS1* (d’Ydewalle et al., 2017), *BDNF-AS* (Modarresi et al., 2012), and others. Moreover, the observation that stable G-quadruplexes are present at the G-rich region of *C9orf72* repeat RNA suggests a link between these motifs and neurodegeneration (Cammas and Millevoi, 2017). Several groups are currently exploring the potential of small molecules to modulate the function of lncRNAs, including some lncRNAs previously implicated in neurological disorders such as *BDNF-AS*, *HOTAIR*, *NEAT1* or *MALAT1* (Pedram Fatemi et al., 2015; Donlic et al., 2018; Abulwerdi et al., 2019; Ren et al., 2019; Simko et al., 2020; Khaled et al., 2021). While the field of RNA-targeting small molecules is still in its infancy, efforts toward understanding the fundamental dynamics between small molecule and lncRNAs recognition together with methodology development will likely contribute to unlock the full potential of using these compounds to treat complex brain disorders by targeting lncRNAs.

Although still early, it has been shown that small molecules can target several classes of RNA besides lncRNAs, including *miRNAs*, repeat expansion regions, mRNAs encoding for intrinsically disordered proteins, splicing modifiers and motifs located at the 5′- and 3′- untranslated regions (5′- and 3′- UTRs, respectively) (Meyer et al., 2020; Yu et al., 2020). Recently, *Risdiplam* received FDA approval for the treatment of SMA (U.S. Food & Drug Administration, 2020), and *Branaplam* is undergoing clinical trials as a therapy for the same disease as well as a for HD (ClinicalTrials. gov, 2021). Both *Risdiplam* and *Branaplam* increase SMN protein levels by acting as *SMN2* splicing modulators (Meyer et al., 2020). Other small molecules have been identified as potential therapeutic agents for neurological disorders but are still in early discovery stages. These include *Synucleozid* for PD (Zhang et al., 2020), *Mitoxantrone* for primary tauopathies (Zheng et al., 2009), and at least one compound with the ability to target expanded repeat regions of *FMR1* mRNA and *C9orf72* mRNA, involved in FXTAS and c9FTD/ALS, respectively (Colak et al., 2014; Su et al., 2014; Wang et al., 2019).

## Conclusion and Future Perspectives

Supported by their characteristics including high cell- and specificity and dynamic regulation of specific cellular pathways at the transcriptional and post-transcriptional level, lncRNA-based therapies could bring important clinical advantages. These include their superior potential as new targets, reduced toxic effects derived from off-target mechanisms, and the possibility to have a biologically meaningful effect with lower compound doses due to their lack of translation, fast turnover, and general low expression levels (Barry, 2014; Bonetti and Carninci, 2017). The fact that most disease-linked single-nucleotide polymorphisms have been shown to map to the non-coding genome further emphasizes the point that exploring non-coding loci can be relevant to identify new potential therapeutic targets (Tak and Farnham, 2015).

However, there are still some challenges to overcome in the field of lncRNAs research. First and foremost, more basic research is needed to address the exact roles of lncRNAs in the brain and identify their related disease-relevant signaling pathways. Second, and related to the first point, further investigation is needed to develop therapeutic strategies that efficiently alter lncRNA transcript levels or repress their function(s) using ASOs and siRNAs provided that the delivery of these molecules to the CNS is tackled. Though impressive progress occurred in the past years regarding the use of oligonucleotide-based therapies, a deeper appreciation of lncRNA structural features and their interactions with DNA, RNA and proteins will open the exciting possibility of targeting these RNAs using small molecules with unprecedented specificity as agonists or antagonists. On the long term, development of orally available, brain-penetrant lncRNA-targeting small molecules could represent a new therapeutic modality for genomic imprinting diseases, neurodevelopmental and neurodegenerative diseases. As new RNA-targeted therapeutic strategies continue to emerge, we believe that unraveling the functional roles of lncRNAs will pave the way to transform lncRNAs originally perceived as “junk” DNA to a therapeutic treasure for patients affected by CNS diseases.

## Author Contributions

RP, AS, and Cd’Y were responsible for the conception of the article. RP did the scientific literature review, created the figures, and wrote the first draft of the review. Cd’Y contributed to the scientific literature review. AS and Cd’Y critically revised the entire review. All authors approved the final version.

## Conflict of Interest

Cd’Y was employed by company Janssen Pharmaceutica N.V. The remaining authors declare that the research was conducted in the absence of any commercial or financial relationships that could be construed as a potential conflict of interest.
